# Perceptions and experiences of living with and providing care for multimorbidity: A qualitative interview study

**DOI:** 10.1177/26335565241240820

**Published:** 2024-03-24

**Authors:** Glenn Simpson, Leanne Morrison, Miriam Santer, Marisza Hijryana, Andrew Farmer, Hajira Dambha-Miller

**Affiliations:** 1 Primary Care Research Centre, 12211 University of Southampton, Southampton, UK; 2 School of Psychology, 12211 University of Southampton, Southampton, UK; 3 Institute of Epidemiology and Health Care, 4919 University College London (UCL), London, UK; 4 Nuffield Department of Primary Care Health Sciences, 6396 University of Oxford, Oxford, UK

**Keywords:** Multimorbidity, interview study, integrated care, personalised care

## Abstract

**Background:**

Experiences of living with and seeking care for multimorbidity is a relatively under-researched field. By analysing experiences of people with multimorbidity, caregivers and care professionals, we can better understand the complex care needs of those with multimorbidity and identify improvements to care management. This paper reports findings from research that elicited the views of key stakeholders to inform future care practice and policy.

**Aim:**

To elicit care recipient and care provider views to understand the care needs of those living with and seeking care for multimorbidity.

**Method:**

A qualitative interview study using purposive sampling of those living with and providing care in multimorbidity.

**Results:**

Increased support to those with multimorbidity and caregivers to navigate care systems was advocated. Establishing trusting care relationships featured prominently in participants accounts. Fragmented care, inadequate coordination and poor communication between care providers, were identified as system-wide challenges. There was agreement that integrated care models were needed, which delivered personalised care, such as shared decision-making, choice in care options and accessing services, and individualised care plans.

**Conclusion:**

We found significant agreement among stakeholders on care need and management in multimorbidity. Understanding the experiences of those with multimorbidity, caregivers and care professionals, can inform future improvements in care management.

## Introduction

Multimorbidity is biomedically defined as the concurrence of two or more chronic long-term conditions in an individual, which can present as a combination of ‘physical and/or mental health’ conditions.^1^ The complex multifactorial aetiology of multimorbidity often makes care management more challenging than with single diseases, leading to increased levels of service usage and higher costs to both healthcare and social care providers.^2-4^

Demographically, multimorbidity is associated with ageing, being more common amongst people aged 65 and above in the UK and internationally.^4,5^ Multimorbidity has higher prevalence in certain populations, including females and individuals with intellectual disabilities.^6,7^ It is “socially patterned, occurring more often and at an earlier age in patients of lower socioeconomic status”, including people of working age.^8^ There is a spatial dimension to prevalence, with individuals residing in areas of relative socio-economic deprivation more likely to experience multimorbidity.^6,9^ Therefore, people’s experiences of living with multimorbidity, and their health trajectories and outcomes, are influenced by wider social determinants of health, not only biomedical factors.^6,8,9^ Consequently, effective care management of multimorbidity requires a holistic and coordinated care approach, often requiring a combination of both clinical and non-clinical interventions.^10^

Experiences of living with and seeking care for multimorbidity remains a relatively under-researched field,^10^ as previous studies have mainly focused on single conditions and/or specific stakeholder groups.^11-14^ While many people with multimorbidity are reliant on caregivers for care and support, the literature relating to caregiving for those with multimorbidity is limited.^15^ As Spiers et al^11^ observe, ‘there is a substantial gap in our understanding of how multimorbidity impacts on the people who live with it, and their carers’. Whilst there is a growing literature on the provision of healthcare to people with multimorbidity, limited evidence is available about the experiences of non-clinical professionals providing care to this cohort, such as practitioners in social services and community care organisations.

This qualitative interview study comprehensively examines how the complex care needs of those with multimorbidity are experienced, by integrating the views of people with multimorbidity, caregivers, and professional care providers in a single analysis. Findings identify possible improvements to care management^3^ and inform future practice and policy in this area of care.

## Methods

### Design

A qualitative study using semi-structured interviews involving people living with multimorbidity and caregivers and care professionals who provide care for this cohort.

### Recruitment and sampling

An iterative recruitment approach was used. We used purposive sampling, to target groups of interest from which to recruit participants to our study. We recruited primary, secondary care and social care professionals, third sector care providers, researchers, people with multimorbidity and informal caregivers. This sampling strategy allowed us to collect the necessary data from a sample most relevant to our research objectives and capture a diverse range of participant perspectives. Forty-five people initially expressed an interest in the study via adverts on social media, specialist online websites (National Institute of Health Research’s People in Research website), newsletters, and use of the snowball sampling technique during the interview process (Table 1). From these initial expressions of interest, 29 individuals agreed to participate in the study. An email invitation containing a description of the study and online access to the participant information sheet and consent form was sent out to those who agreed to participate. Participants were over 18 years old, able to provide consent to study participation and communicate in the English language.

**Table 1.** Advert used to recruit study participants via specialist online websites and newsletters, and social media.

**Website Advert**
Have you had experience of multiple health conditions? You might have received treatment or other support yourself for these conditions, been a carer or a relative of someone who has lived with multiple health conditions, or you could be a professional who has worked with this group of patients. If so, we are interested in your views!
We are carrying out a study to explore new ways of treating and supporting both the medical and non-medical needs of people with multiple health conditions.
We’d like to interview you on the telephone or video-link for between 30-60 minutes, at a convenient time for you, to ask about your experiences and views of multiple health conditions.
If you are interested or have any questions, please email us at: G.W.Simpson@soton.ac.uk
**Social Media Advert**
Have you had experience of multiple health conditions?
We are carrying out a study to explore new ways of helping people with these conditions. Our researchers will carry out a telephone or video-link interview for 30-60 minutes to ask about your views. Interested?
Please email us at: G.W.Simpson@soton.ac.uk.

### Data collection

Participants were interviewed by telephone or video according to their preferences. Interviews were conducted by GS, HDM and MH, between February and May 2022, each lasting between 30-60 minutes. An interview schedule was developed with a focus on understanding the perspectives of those with multimorbidity, caregivers and care professionals to gain insights into the key clinical and non-clinical care needs of people with multimorbidity (Table 2). While the same questions were discussed in all interviews, we adopted a flexible approach to ensure that participants were able to raise any related subject of importance. Interviews were recorded and transcribed verbatim. Transcripts were anonymised with an assigned study number.

**Table 2.** Sample interview questions for participants.

i) Interview script care professional’s version - for healthcare, social care, and allied/associated professionals			
Question number	Main Questions	Prompt Questions	Probes
1	**Overview:** • **Healthcare professional’s background**: Please tell me about both your role and experience of treating or supporting people with multiple long term health conditions or multimorbidity? • **Social care and other related professional groups**: Please tell me about your role and experience in supporting people with multimorbidity?	• What specific service, treatment or support do you provide to people with multimorbidity? • Can you tell me about any services/treatment/support (as applicable) you provide for long-term physical conditions? • Can you tell me about any services/treatment/support (as applicable) you provide for long-term mental health conditions?	
2	What are the *key factors* that you think may influence people’s experiences of living with multimorbidity?	**•** What are key individual barriers influencing the experiences of people living with multimorbidity? **•** What are key social barriers influencing the experiences of people living with multimorbidity? • What do you think are the physical and mental health barriers influencing the experiences of people living with multimorbidity? • What are key institutional barriers, or barriers within services, influencing the experiences of people living with multimorbidity?	**Probe topics for individual and social barriers:** • Individual lifestyle factors which include their behaviours such as smoking, alcohol use, and physical activity. • Social and community networks include family and wider social circles. • Living and working conditions include access, barriers, and opportunities in relation to jobs, housing, health care services, education and welfare services, water and sanitation, home or work environment. • General socioeconomic, cultural and environmental conditions include factors such as disposable income, taxation, availability of work, and language. • Individuals’ physical surroundings/ environments (e.g., Is it safe and supportive environment? Proximity to health services, proximity to local services (post office, supermarkets, etc.)). • Availability and accessibility of public transport. **Probe topics for medical and institutional barriers:** • Lack of continuity in treatments and supervision for people with multimorbidity. • Medication adherence. • Challenges of sharing records between organisations. • Poor communication between and within organisations. • Lack of coordination between health professionals or organisations. • Organisations focusing on their own specialist interests (organisations protecting their interests and retaining control over specialism).
3	What are the main factors that are key to *improving* care to address the *medical needs* of people living with multimorbidity?		• More personalised, whole person care. • Access to reablement. • Integrated care approach that is tailored to the individual and his/her environment. • Improving patients’ self-management. • Pro-active care with early treatment of risk factors. • Treatment interaction. • Ensuring continuity of care. • Effective communication between health providers and patients. • More key individuals/teams as interface roles to bridge gaps between service providers. • Co-location to bring professionals together from multidisciplinary backgrounds.
4	What are the main factors that are key to improving care to address *the social needs* of people living with multimorbidity?		• More personalised, whole person care. • Support for social isolation. • Access to reablement. • Integrated care approach that is tailored to the individual and his/her environment. • Improving patients’ self-management • Educating informal caregivers and patients • Effective communication between health providers and patients. • Information for patients and caregivers regarding integrated care they receive and how it works.
5	What do you think about holistic, personalised or whole-person care?	• What are the facilitators to the holistic and personalised or whole person-care? • What are the barriers to the holistic and personalised or whole person-care? • Do you feel that the treatments and care have been integrated/joined up for people with multimorbidity?	

ii) Interview script, lay person version ‐ for those living with multimorbidity and informal caregivers			
Question number	Main Questions	Prompt Questions	Probes
1	**Overview:** **People with Multimorbidity:** Please tell me about what it is like living with several different long-term health conditions? **Caregivers:** Please tell me about what it is like to support people with several different long-term health conditions?	• Do you mind telling me what conditions you have? We are interested in both physical and mental health conditions. (NB: Please only share with us the details that you are comfortable with). You do not have to share the detail of your health if you do not want to. • What kinds of services/ treatment/social care support you have used as a result of your long-term physical conditions? • Describe the kinds of services/treatment/ support you have received for your mental health?	• If participants are willing to open up about different conditions they have (physical and/or mental health), probe further around the differences or similarities in their experience of the conditions.
2	**People with Multimorbidity:** What daily living and lifestyle challenges do you experience as a result of your long-term health conditions? **Caregivers:** What are daily challenges do you experience when you are taking care of people with long-term health conditions?	• Is there anything that stops you from doing what you would like to do day-to-day, related to your health conditions? (NB: Non-medical and social needs are the main focus here). **Caregivers:** • What challenges are being experienced by the person(s) you have supported and provided care for?	• Individual ‘lifestyle factors which include their behaviours such as smoking, alcohol use, and physical activity. • Social and community networks include family and wider social circles. For example: availability of family members or friends who can assist as informal caregivers, availability of respite care for caregivers. • Living and working conditions include access, barriers, and opportunities in relation to jobs, housing, health care services, education and welfare services, water and sanitation, house or work environment.
3	**People with Multimorbidity:** What barriers or challenges have you experienced with access to treatment, and healthcare services you need for your health conditions? **Caregivers:** What barriers or challenges have you experienced when helping people with long-term conditions access their treatment and healthcare services?	• Is there anything that stops you from getting the treatment or help that you need for your health condition, or that makes this difficult? (NB: Medical and healthcare challenges are the main focus here).	• Social and community networks include family and wider social circles. • General socioeconomic, cultural and environmental conditions, including factors such as disposable income, taxation, availability of work, and language. • Individuals’ physical surroundings/ environments (e.g., Is it safe and supportive environment? Proximity to health services, proximity to local services (post office, supermarkets, etc)). • Availability and accessibility of public transport.
4	**People with Multimorbidity:** Are there any additional services or support that would help to improve your quality of life? **Caregivers:** In relation to the person(s) you care for - Are there any additional services or support that you think may help to improve their quality of life?	• Are there any treatments or support you think may improve your health conditions? • What support or assistance could more effectively support your day-to-day life and improve your experience of living with the health conditions?	• More personalised, whole person care. • Support for social isolation. • Access to reablement/physio/OT/wheelchair or other aids. • Integrated care approach that is tailored to the individual and his/her environment. • Improving patients’ self-management. • Pro-active care with early treatment of risk factors. • Treatment interaction. • Educating informal caregivers and patients. • Ensuring continuity of care. • Information for patients and caregivers regarding integrated care they receive and how it works.

### Data analysis

We used inductive reflexive thematic analysis.^16^ The first stage involved the research team gaining familiarity with the data through repeated reading of transcripts. A sample of transcripts from the first few interviews was coded by MH and discussed within the team for initial analysis and interpretation (GS, MS, LM), until consensus was reached leading to the formulation of a coding framework. Subsequent rounds of coding led to further iterative refinement of the framework. We identified recurring patterns in the data leading to the development of themes. Throughout the analytical process, a form of constant comparative analysis was used to identify key differences or similarities in the data. QSR NVivo software (version 12) was used to manage the data and the Consolidated Criteria for Reporting Qualitative Research (COREQ) checklist guided reporting.

## Results

We interviewed 29 participants: 15 females and 14 males (Table 3). Thirteen experienced multimorbidity and/or were caregivers. There were 16 professionals from various care sectors and UK regions (Table 3). Our thematic findings are discussed below. Supporting evidence is provided through illustrative quotes from the study participants.

**Table 3.** Study participant characteristics.

Study Number	Age group	Gender	Occupation/Role	Ethnicity	UK nation	Region
1	50-59	Male	Social services professional, local government	British	England	South West England
2	40-49	Male	Living with multimorbidity	Any other White	England	West Midlands
3	60-69	Female	Social services professional, local government	Any other White	England	South East England
4	70-79	Male	Living with multimorbidity/Caregiver	British	England	North West England
5	40-49	Female	Living with multimorbidity	British	England	South West England
6	70-79	Female	Caregiver to an individual with multimorbidity	British	England	South West England
7	60-69	Male	Primary care professional and clinical academic	Irish	Scotland	Fife
8	30-39	Male	Primary care professional and clinical academic	British	Scotland	Tayside
9	40-49	Male	Support Coordinator, for a housing association (Registered Charity)	British	England	South East England
10	60-69	Female	Secondary Care, Registered NHS Nurse	British	England	North East England
11	40-49	Female	Living with multimorbidity	Any other White	England	South East England
12	30-39	Female	Care Manager, private health provider and living with multimorbidity	British	England	East of England
13	18-29	Male	Academic researcher in multimorbidity	British	England	North West England
14	30-39	Male	Secondary care Specialist Clinician and Clinical Research Fellow	British	Scotland	Scotland
15	50-59	Female	Living with multimorbidity	Irish	England	South East England
16	40-49	Male	Academic researcher in multimorbidity	British	England	North West England
17	40-49	Female	Community Care Assistant, private health and social care provider	British	England	East of England
18	40-49	Female	Primary care professional	Other	England	South England
19	40-49	Male	Social services professional, local government	White/Black Caribbean	England	South East England
20	50-59	Male	Academic researcher in multimorbidity	British	England	Greater London
21	50-59	Female	Primary care professional	British	England	South East England
22	50-59	Female	Training and Development Co-ordinator (Long-term conditions), advice and support organisation for people with LTC and disabilities (Registered Charity)	British	England	South West England
23	80+	Male	Living with multimorbidity	British	England	South East England
24	60-69	Female	Living with multimorbidity	British	England	North West England
25	18-29	Male	Caregiver to an individual with multimorbidity	Bangladeshi	England	Greater London
26	60-69	Male	Caregiver to an individual with multimorbidity	British Asian	England	East Midlands
27	70-79	Female	Living with multimorbidity	British	England	North East England
28	70-79	Female	Living with multimorbidity	British	England	South East England
29	40-49	Female	Living with multimorbidity	British	England	West Midlands

## Experiences of people with multimorbidity

### Daily challenges of living with multimorbidity

Participants recounted the breadth of practical and emotional impacts that they attributed to the challenges of living with multimorbidity. These appeared to shape multiple decisions in everyday life, including work, leisure, domestic, social and financial considerations:

…it affects every aspect of your life. It affects what we eat, where we go, where we go on holiday (Person with multimorbidity, SN24).

Ten o'clock in the morning is the earliest I can do, for outside things, because I have my medication… and they have to be the first thing ...If I ran the house, I wouldn't be able to do anything else (Person with multimorbidity, SN5).

Travelling has been a nightmare… I haven't flown anywhere for the last ten years because I just can't risk that. The insurance costs. Yes, it's basically that freedom… 'Let's go here today and forget about anything else' (Person with multimorbidity, SN11).

Participants spoke about not only the practical impacts of multimorbidity, but the limitations to everyday activities brought about by fatigue.

…one of the most severe symptoms is constant fatigue …That limits me in a lot of ways, because I've only got so much energy …so the only time I get to go out is to go shopping. (Person with multimorbidity/Caregiver, SN4)

Emotional and psychological experiences of those seeking care featured prominently in participants accounts, as did recognition that the practical limitations resulting from multimorbidity bore relation to an individual’s quality of life and sense of wellbeing:

I was quite an active man. …I used to play rugby, and then as I got older, I started playing golf. I was never any good at it, but at least I enjoyed playing a round of golf ...I miss that. (Person with multimorbidity/Caregiver, SN4).

It happens sometimes that I have a chance to reflect …’oh it's all so unfair’. I've had a pretty healthy lifestyle. I've never done drugs. I've never abused alcohol, etc. Still, I am ill. Sicker than most of the people that I know (Person with multimorbidity, SN2).

### Challenges of accessing and using care

Delays and long waiting times to access services were common experiences, both for physical and mental health concerns. Participants found this both frustrating and worrying, in some cases feeling that it could affect disease progression or that it left them experiencing symptoms, such as pain, for longer than they should:

…they will look at my spinal injury, and then I'll say that I'm having problems with my mental health, and they'll say: 'Okay, so that's obviously a separate condition.' So, then they'll look at referrals for that. By the time that referrals come through, that's two months down the line and things have progressed. Something else could be a problem then. It's just too long to access these services. (Care Manager/Person with multimorbidity, SN12)

Similar challenges were experienced by another participant when seeking mental health care:

I tried to access mental health care because I was in a situation where I just felt really overrun with everything and I was suffering anxiety …I was referred to IAPT's. It took them six months to offer me an appointment. (Person with multimorbidity, SN11)

Another participant also found that the responsiveness of service providers was too slow to address their immediate care needs:

If I had called the rheumatology... They can't suddenly prescribe a medication that I need really quickly when I'm in that much pain ...When you phone, they encourage you not to leave a message …unless you can't email, and then they say: ‘email them’ ...then it says: 'We'll come back to you in four days'. It isn't very helpful when you need something quickly. (Person with multimorbidity, SN 29)

Some participants experienced difficulties accessing care due to transport and travel difficulties, particularly for multiple appointments:

I couldn't get to the hospital and when I asked for help with transport, they keep referring me to different places. …Then I told them: ‘listen, unless you sort that out, I won't be able to come’. …I think I paid like maybe £80 to get to hospital. (Person with multimorbidity, SN2)

The accounts of several participants indicated that difficulties accessing services were related to a care system primarily designed to treat single conditions:

...you only have time to tell them the one thing that's wrong with you, and I've gone in with three things that are wrong with me because over time, the one thing has caused another thing, which has caused another thing, and also in the meantime, something else has cropped up. …This is beside my desk, which is a list of all the things that I need to talk to the doctor about. …I can't deal with all of those things at once. (Person with multimorbidity, SN15)

A participant described the challenges of care coordination between primary and secondary care. This required a proactive response on behalf of the participant, which involved them having to coordinate and self-manage aspects of their care regime.

…GPs and hospitals, they could actually work together. …I often find that I'm the patient and I'm doing the coordinating. ...They'll say: 'What's so and so thing?' I'm like: 'Hang on. I'm just the patient. You sort it out.’ At one point, I've had three or four consultants all saying different things. (Person with multimorbidity, SN5)

Another participant had a similar experience, but further articulated that, although able to be their own ‘care coordinator’ when well, they did not feel able to do this when unwell:

I have experienced a lot of problems trying to get a holistic approach to planning my healthcare and treatment. I find that the different specialisms almost work in silos. They will communicate by letter, but the admin is so poor that often the letters are not timely or don't arrive at all. There are very few doctors I know who will actually talk to you and say: 'Right, I'm going to sort this out now …If you're caught up in the middle of that and like me, you're reasonably able to deal with it, you end up becoming the coordinator of all your healthcare. …that's okay if you're feeling well, but if you're going through a bad patch, and you're not really able to speak coherently …then you'll feel very vulnerable. (Person with multimorbidity, SN24)

A participant described how difficulties with care coordination affected their ‘choice of care’ and the ways in which learning to navigate different health services in different regions caused an additional burden on them:

It was more complicated getting the GP to do a blood test in O* to then send it to C*. It just didn't happen... I'd have preferred to keep my care in C*. There wasn't any issue with keeping it there in the sense that I felt like I was getting the best care possible there but the impact of me trying to coordinate bloods ...was just so much hassle, that I was forced to basically transfer my care across to O*. It takes that option of choice of care away. (Person with multimorbidity, SN11)

### The need for continuity and a holistic approach to care

The need for a holistic approach to care was emphasised:

One GP I have does that, but he only works part-time and it's really hard to get an appointment with him, but he will look at the big picture... I have a huge list of medications ...and he will look at that on a holistic level as opposed to one thing at a time, but most of the time it's impossible to get an appointment with him, and I end up just dealing with one person about one thing. (Person with multimorbidity, SN15)

Care continuity was valued by those living with multimorbidity, especially in relation to establishing trusting relationships with the same care professionals:

People with long-term health conditions build up a relationship with the staff. So, if you haven't got that …you've got to start off from square one, it's very difficult, whatever your condition. (Person with multimorbidity, SN24)

## Caregiver’s experiences

Caregiver’s accounts of impact on themselves of caring for someone with multimorbidity, the breadth of limitations on everyday life and subsequent emotional impact, bore similarities to the accounts of people with multimorbidity.

### The demands of caregiving and coping

The unpredictable nature and trajectory of long-term health conditions made caregiving a daily challenge:

...because [of my daughter’s] condition, we never know from one day to the next, almost, how she's going to be. It's a bit like living on a knife-edge. ...She can be okay and then in half an hour/an hour, we have to be calling an ambulance. (Caregiver, SN6)

…it has impact on …my well-being. Trying to maintain the house, trying to maintain the family life, trying to maintain the job, so it was very difficult to do the balancing act, initially …it's just finding your own pathway, basically. (Caregiver/Carer’s group representative, SN26)

Similarly, some caregivers spoke about the biographical adjustments or ‘acceptance’ of limitations when caring for someone with a combination of physical and mental health conditions.

…if I'm going out, for example, I've either got to take C* with me, or I've got to make sure there's a carer for him. So, as far as my social life is concerned, yes, I have to think about that, but I've been doing it for a long time, and so it's just habitual now. It's just what I do. Carers get on with it (Person with multimorbidity/Caregiver, SN4).

### Continuity of care

Others also highlighted the importance of continuity of care from the same care team:

I was fortunate. ...in terms of medical professionals, I had the same people. So, the continuation of care was there. (Caregiver, SN26)

Caregivers often performed a critical role in providing practical and emotional support, as well as assisting those to whom they provided care, to navigate the complexities of the care system and perform a ‘care coordinator’ role where the person they were caring for was less able to do so themselves. Caregivers believed their inputs were important to optimising care management and expressed a desire to engage in a collaborative care approach with care professionals.

## Care professional’s experiences

### Complex care needs

Care professionals highlighted the inherent complexities of providing care to those with multimorbidity. A GP explained:

I had a patient with a swollen foot this morning. He does have gout, so that's the obvious problem, but he's also undergoing radiotherapy for prostate cancer, and he's hypertensive and he's being treated for depression. You're trying to work out the Stott and Davis model of the consultation. You have to deal, first of all, with presenting conflicts. If you don't do that, then patients are never going to pay any attention. Then you go on to other things, like dealing with their established other conditions - and that's multimorbidity - and then, potentially, onto the other parts of the consultation which are health promotion-type activities. (Primary Care, SN7)

### Limitations within the care system

Capacity and resource constraints across the care system, featured prominently in care professional’s accounts. In social care, the challenge of recruiting and retaining skilled care personnel with the requisite skills were highlighted:

My concern in social care is we're losing social workers by the day …and I know they say grow your own and bring new people in, but … people just don't want the grief of it. (Social Care Manager, SN19)

Clinicians identified the challenges of providing care within the limitations imposed on practice by the operational realities of the care system, which contributed to ‘fragmented care’:

I see it as fragmented healthcare, the way that it's all focused in terms of …the specialty lens …the ability of patients to actually have informed information about their conditions, often is limited because it's often you've got ten minutes, and you say: ‘well, you should probably lose a bit of weight rather than actually say how can they do that, what's locally available.’ That provision of information is often lacking... All these factors then limit what patients can take from these consultations, because you're focusing on the issue at hand. I see those as the fundamental difficulties. A lot of it is fragmented healthcare, secondary care-based challenges. (Primary Care, SN8)

Other care professionals reported similar difficulties experienced by people with multimorbidity and caregivers:

They [people with multimorbidity] don't really know who they're seeing, and what that person's role is in relation to their care, and therefore, what questions they can take to that professional. Obviously, they would get much more out of their 15-minute appointment …if they knew what that person could do for them, and what to ask them. (Health and Wellbeing Coach, SN18)

I think to navigate the system, you need to have a good understanding of the system! If you've got a mental health condition and you're not in good shape physically, it's kind of difficult to do that … Then that really falls on the family to do that, and to chase up… (Social Services, SN1)

### Communication challenges

Poor communication and inadequate processes for exchanging patient information between care providers were a common concern, although care professionals acknowledged this was a difficult challenge to address:

Having come from a business background, I am appalled at how departments do not communicate with each other. …the need for efficient communication between different stakeholders, it's very important. It's difficult because different agencies have different agendas, different priorities. Sometimes those priorities clash. …that's a difficult barrier to overcome. (Voluntary Sector, SN9)

### Patient and care professional relationships

Care professionals recognised the importance of establishing trusting relationships with patients as critical to good practice and care delivery:

…the patient has to be consulting someone that they trust, and that the clinician puts themselves in a position to make an informed assessment of the patient's problems; and then discuss potential solutions to whatever the problems are. (Primary Care, SN7)

## Perspectives on improving care

A number of common themes to improve care in multimorbidity were identified. Care recipients and care providers stressed the need for both holistic and personalised approaches to improve care management of the range of clinical and non-clinical needs associated with multimorbidity:

…when you have a long-term disease, a few of them, it's really important to be treated holistically, to treat your mental health, …to have someone help you with diet, with exercise, physiotherapy, etc. (Person with multimorbidity, SN2)

…the NHS is …sometimes not looking at preventative, and just treating symptoms. We're looking at symptom management, so they've got pain; painkillers …why are they suffering with pain? Can anything be done about it? How can we think a bit more holistically… looking at a whole range of treatments, physiotherapy, whatever it is. (Social Services, SN1)

People with multimorbidity highlighted the need for increased availability and access to ‘social’ or non-clinical support to improve psychosocial outcomes and mental wellbeing more generally:

…just being given more choice to a better degree of understanding of what support and resources are available beyond the healthcare sector. So, for example, what else can they [people with multimorbidity] turn to which may have say a positive impact on their life …in other fields or areas that they might …look at taking an interest in. (Person with multimorbidity, SN25)

People living with multimorbidity and care professionals identified the social prescribing model as an effective intervention, which could be extended to strengthen the delivery of non-clinical care support:

…it's that whole social prescribing because people do find it difficult to access GP and medical support. I think when they're there, they're very conscious of their allotted time (Person with multimorbidity, SN24).

…normal models that we were using, so social prescribing, it was great being able to prescribe swimming classes, exercise classes for deprived patients with long-term conditions. That all just went. (Primary Care, SN8)

All participants advocated greater efficacy in communication between care sectors as an essential component to drive care improvement in multimorbidity:

I think hospitals need to have …better, clear communication dialogue with the GPs, then we'd know what the action plan is. The patient would know, they don't have to chase it up. (Caregiver, SN26)

I'd say probably [the] relationship between the GP and hospital could be better. Again, I think it depends on which GP you end up speaking to. (Person with multimorbidity, SN29)

Some participants identified that care coordinators, who act as an ‘interface’ between patients and care professionals, have potential to enhance coordination of care:^17^

…care coordinators in the NHS; since that role has been created two or three years ago, they are in a perfect position to take up that role [supporting patients at consultations], because they already coordinate patients care, and the multiple complex areas [where the] …patient may have needs. (Primary Care, SN18)

Having a dedicated ‘continuity’ social worker, could be an effective role in supporting continuity of care among those receiving social care:

…people …want to have a social worker that carried on working with them. You've got that relationship with that person …who you trust, who's going to be there for you... sometimes people feel a bit alone in the system in that way. From a health or a social care point of view, I think having a worker. (Social Services, SN1)

There was consensus among participants that integrated or joined-up care models were key to long-term improvement in care for multimorbidity:

I think we need a different model, …it has been working in some of the doctor's surgeries who have been opening at irregular times... as social care …we need to adopt a similar approach where we're a bit more available when the users are available really …so an integrated care model is what I'm essentially saying. (Social Services, SN19)

…in terms of improvement …there has been issues where there's not joined-up working, where health won't talk to the social care and vice versa, which has had impact on quality of care. (Caregiver, SN26)

## Discussion

This study explored the care needs of those living with and providing care for multimorbidity. Our study found a high degree of convergence between the perspectives of the different stakeholders, indicating there is shared agreement on both the key care needs of those with multimorbidity and possible future improvements to care management. All participants emphasised the value of adopting a holistic and personalised approach to care that considers not only clinical and social care needs but also wider social determinants of health and wellbeing. Increased support to patients and their caregivers to navigate care systems more easily and to speed-up or streamline diagnosis and treatment, was widely advocated. This could involve an enhanced role given to care navigators/coordinators or primary care link-workers, as well as social care providers exploring practical ways of improving continuity of care for service users/clients with dedicated social worker support.

Establishing trusting patient and care professional relationships, featured prominently in participants’ accounts. Fragmented care, inadequate care coordination and poor communication between care providers, were identified as system-wide barriers that needed to be overcome to improve service delivery and care outcomes in multimorbidity. To address these barriers, there was common agreement that integrated care models were needed, which enable closer collaboration among care providers to facilitate joined-up frontline care delivery as well as supporting ongoing continuity of care to people living with multimorbidity.

Those with multimorbidity shared experiences of traversing a complex healthcare journey, including managing care regimes, coping with symptoms and life changes, which contributed to a reduced quality of life. This burden took an emotional toll. People with multimorbidity encountered challenges accessing services, especially delays in referrals and treatment. Travel barriers caused difficulties in access to, and choice of, care. The importance of effective communication with care professionals was highlighted, not only to enable patients to be more involved in care decisions but also establishing trusting relationships with care professionals.

Caregivers often performed a critical role in providing practical assistance with activities of daily living, emotional support, as well as assisting those to whom they provided care, to access and ‘navigate’ the complexities of the care system. In this context, caregivers stressed the importance of forming trusting relationships between themselves, the care recipient and care professionals. Caregivers frequently cited the burden of caregiving and the impact this had on their lives. A common concern expressed was the lack of provision or advice available to support caregivers in their role.

Care professionals identified challenges associated with assessment and management of people with multimorbidity. Communication was highlighted as a critical factor in care practice and delivery, including the need for improvements in exchanging information and records between care sectors. Care professionals reported challenges of providing complex care to people with multimorbidity in a care system characterised as experiencing capacity constraints, especially in social care, where recruitment and retention of staff, in particular, remains problematic.^10^ Other systemic challenges were identified, including a care system designed around management of single conditions, a model which care professionals acknowledged does not effectively optimise care for people with multimorbidity.

### Comparison with the literature

To our knowledge, there have been a limited number of studies examining the care needs of those with multimorbidity from a comprehensive range of stakeholder perspectives.

The perspectives of people with multimorbidity have been documented in earlier work, including the need for a holistic approach to care, continuity of care and establishing trusting relationships with care professionals.^18,19^ Other sources have identified delays in appointments and referrals experienced by people with multimorbidity, which can be a significant factor influencing care outcomes.^19^ Further, studies have found that those with multimorbidity ‘reported better coordination of care when they had more time with their General Practitioner, which ultimately improved individualised care’.^14,20^ Our results also reflect the findings of other research in that people with multimorbidity stressed the need for timely and convenient ‘access to care when and where they needed it’, which is especially important in preventing further deterioration in health status.^14,21^ Additionally, the significant impact on a patient’s quality of life and wider emotional well-being, and the ‘high symptom burden’ often associated with multimorbidity, are explored in earlier work.^22-24^ Our study contributes to the literature examining the experiences of people with multimorbidity when navigating care systems.^24,25^

The critical role of caregivers is discussed in previous studies, which found caregivers undertook a range of time-intensive and demanding care responsibilities for those with multimorbidity.^26,27^ Existing studies describe how caregivers' quality of life, and their physical and mental well-being can be affected by the burden of caregiving.^28^ However, in the context of multimorbidity, there has been limited research investigating ways of involving caregivers in a more collaborative care approach with care professionals.

Previous research has shown care professionals recognise the constraints of providing care to those with multimorbidity, especially ‘within a fragmented health care system whose policies and structures remain organised around single condition care and specialisation’.^29,30^ There is a literature documenting the challenges experienced by care professionals in implementing care strategies to manage the complex care needs of multimorbidity patients.^31^ The importance of the patient-care professional relationship, as viewed by care professionals, has been explored elsewhere, although mainly from a primary care perspective^32-34^. Research suggests GPs ‘prioritised this relationship above all other aspects of care, as it was perceived to bring benefits to the consultation and treatment outcomes’.^29,30^ Whilst GP/primary care professional perspectives on providing care to those with multimorbidity are described in the literature, there is currently limited research examining the experiences of care professionals in other sectors, including social care. There is a limited literature exploring care professional’s perspectives on the management of both the clinical and/or non-clinical needs of people with multimorbidity.^29,30^

### Strengths and limitations

A strength of our study is the sample breadth, which included a diverse range of participants living with or providing care for people with multimorbidity. This is in addition to sociodemographic and geographical diversity of participants across England and Scotland. We had a reasonable sample size of 29 participants and the reflexive thematic analysis method ensured we adequately captured and summarised views.

Interviews were carried out on the telephone or through video calling and it is plausible that in-person interviews may have elicited more varied discussions. Similarly, our recruitment strategy was heavily reliant on online services (e.g., email study invitations, online advertisement of the study) and all interviews were conducted remotely. This may have biased our sample to those who had access to and were competent with online services. The sample size was small; therefore, our results may have limited transferability. We only recruited participants who communicated in English, which may have made it difficult for under-represented non-English speaking groups to be included in the study.

## Conclusion

Our study found significant alignment in the views of key stakeholders on care need and management in multimorbidity.^35^ Understanding the experiences and insights of those with multimorbidity, caregivers and care professionals, can inform future improvements in care management. Future research could further evaluate potential enhanced roles for care navigators/coordinators or social care providers to explore practical ways of improving continuity of care for service users/clients. Multimorbidity presents complex care challenges requiring a comprehensive systems-wide approach to better address both clinical and non-clinical care needs^10,17^. This requires strategic and operational change to care systems based on collaborative care models that deliver the holistic and person-centred care necessary to improve care outcomes for this cohort^18,36,37^.

## Ethical statement

### Ethical approval

Ethical approval for the study was provided by the Faculty of Medicine Ethics Committee, University Hospital Southampton, (reference number 67953).

## Data Availability

Data are available from authors with a reasonable request.
